# Newborn Penile Trauma in Delayed Breech Presentation: A Case Study

**DOI:** 10.7759/cureus.97843

**Published:** 2025-11-26

**Authors:** Linette Acevedo Iglesias, Michael T Luong, Nichole Cufino, Patrick Wu, Pedro E Montanez, Paul Danahy

**Affiliations:** 1 Family Medicine, AdventHealth Sebring, Sebring, USA; 2 Osteopathic Medicine, Lake Erie College of Osteopathic Medicine, Bradenton, USA; 3 Pediatrics, AdventHealth Sebring, Sebring, USA; 4 Orthopedic Surgery, Lake Erie College of Osteopathic Medicine, Bradenton, USA

**Keywords:** breech presentation, general obgyn, pediatric penile trauma, pediatric reconstructive urology, penile injury

## Abstract

Breech presentation occurs in a small number of term pregnancies and can result in increased neonatal morbidity. Genital trauma, although uncommon, is a possible complication of footling breech presentation, especially when maternal presentation for cesarean section is delayed. One such case is that of a suspected 35-week gestational-age male neonate born via emergency cesarean section for footling breech. The mother had a history of polysubstance abuse and received no prenatal care due to her unawareness of the pregnancy until a delayed presentation at labor and delivery. At delivery, the neonate was seen to have superficial penile trauma with sloughing of the scrotal skin in addition to bruising of the prepuce. Further evaluation confirmed that injuries were limited to the superficial dermis, and deeper genitourinary structures were spared. The neonate’s injuries were treated conservatively with topical bacitracin and supportive care, with complete resolution seen in the neonatal intensive care unit by the seventh day of life. Recovery was complicated by neonatal abstinence syndrome and gram-positive sepsis, both of which were managed appropriately. This case highlights a rare but preventable complication of footling breech presentation. Mechanical compression and friction with uterine contractions during labor likely played a role in the genital injury. Conservative management proved adequate for mild superficial injuries such as this one, and no long-term complications are expected. This case reaffirms the importance of prenatal care. With routine prenatal care, footling breech presentation would have been identified, a cesarean section would have been planned, and mechanical trauma during labor avoided. Early recognition and consistent follow-ups are necessary for uncomplicated deliveries.

## Introduction

Breech presentation in childbirth refers to the initial presentation of lower fetal extremities instead of the fetal head in the birth canal. Following the landmark Term Breech Trial in 2000, published in the Lancet, planned caesarean section has been the standard of care for breech presentations. The study concluded that planned caesarean section is more favorable than planned vaginal birth for the term fetus in the breech presentation, with planned caesarean sections demonstrating statistically significant lower perinatal and neonatal mortalities. Furthermore, cesarean sections and vaginal births demonstrated no differences between groups in terms of maternal morbidity [1].

Breech presentation occurs in approximately 3-4% of term pregnancies in the United States, and its incidence has been increasing over the past few years. According to the National Center for Health Statistics, the number of breech births increased by 61.7% from 2019 to 2022 (n = 423 in 2019, n = 684 in 2022), whereas U.S. births increased by only 33.2%. Current literature has shown that the risk for intrapartum and neonatal morbidity is higher in breech births than in cephalic births [2].

Birth injuries can occur with breech presentations. Perinatal asphyxia may result from breech presentation, leading to mid-to-severe long-term neurological deficits and 30% mortality. Long-term survivors are left with severe sensorineural hearing loss, blindness, seizures, inadequate mental development, and debilitating cerebral palsy [3]. More frequently, frontal bossing, prominent occiput, low-set ears, torticollis, and hip developmental dysplasia are seen with breech presentations due to mechanical compression of fetal body parts [4]. Neonatal hip instability occurs in 12-24% of neonates with a breech presentation. More subtle forms of hip dysplasia that are not detected in childhood and adolescence may contribute to the long-term development of osteoarthritis and an increased need for surgical intervention as an adult [5].

In addition to the more common forms of birth injuries associated with breech presentation, such as hip developmental dysplasia, genital injuries are relatively uncommon but have been reported in various case reports. They range from labial swelling in females to testicular torsion and testicular gangrene if unresolved within six hours of onset in males. Testes are generally non-salvageable by the time of detection. Furthermore, current literature has shown that testicular injury is more likely to occur in babies with a birth weight of greater than or equal to 2,500 grams or born to primiparous mothers. These genital injuries should be carefully examined and managed, as long-term complications, including sterility, may occur [6-7]. Unfortunately, there is currently a lack of research on the long-term complications of penile trauma among neonates born with breech presentation.

## Case presentation

**Figure 1:**
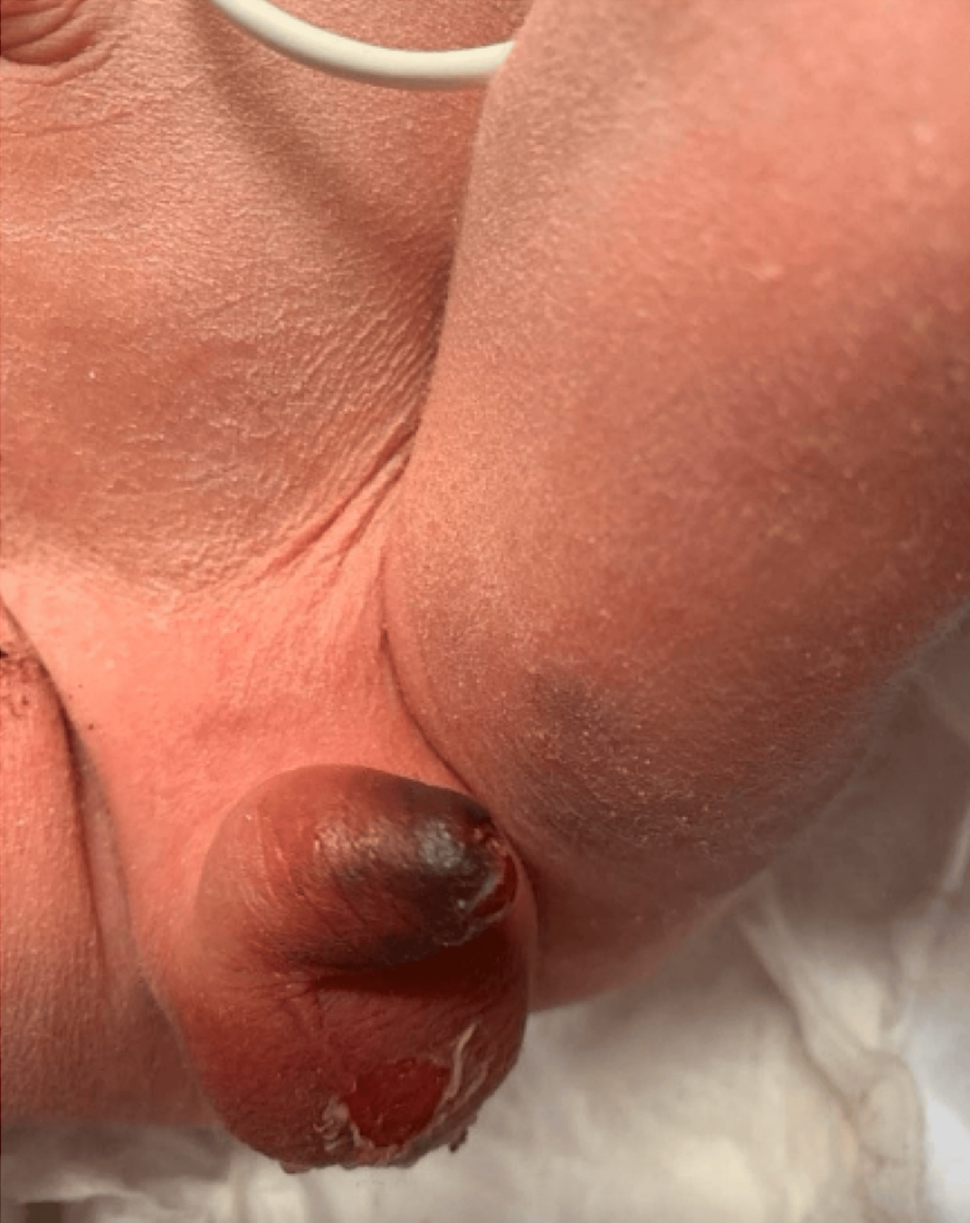
Image depicts the initial evaluation of the neonate shortly after delivery via c-section. Ecchymosis of the prepuce can be seen.

**Figure 2:**
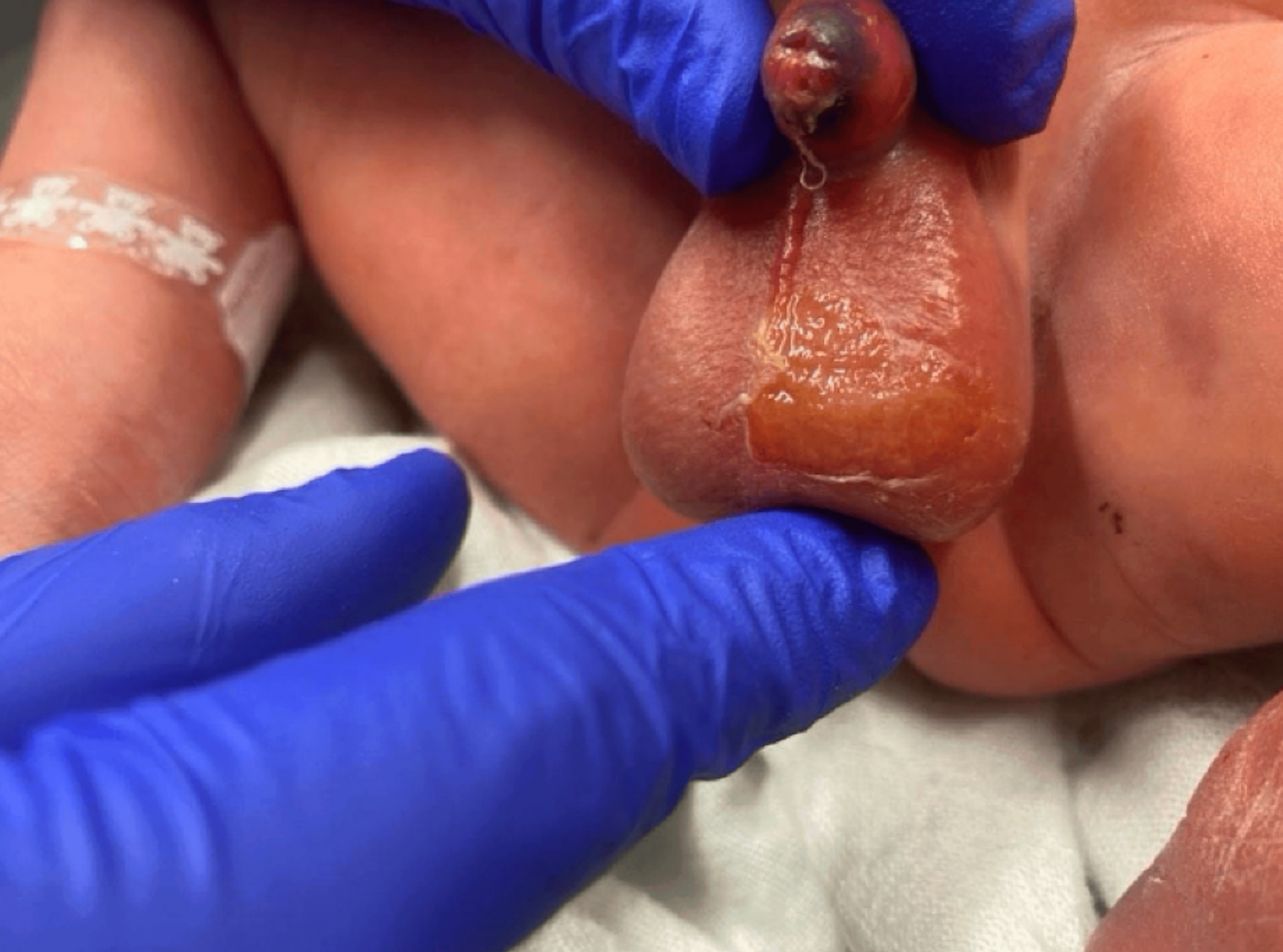
Image depicts the initial evaluation of the neonate shortly after delivery via c-section. Scrotal sloughing of the dermis is seen.

We present the case of a male neonate who was born via emergency cesarean section due to footling breech complicated by delayed presentation and lack of prenatal care. Although the exact gestational age was not documented, the patient appeared to be 35 weeks based on physical examination. At birth, the patient weighed 2,954 grams. The patient's appearance, pulse, grimace, activity, and respiration (APGAR) were scored 7 and 8 at one and five minutes, respectively. Upon delivery of the infant, postnatal examination revealed sloughing of scrotal skin and bruising of the prepuce, as can be seen in Figures 1-2.

Further evaluation did not reveal any fractures or deeper tissue damage. Given the penile injury and concerns about additional trauma, the patient was transferred to the neonatal intensive care unit, with consults placed for pediatric urology for further evaluation and management.

Maternal history

The neonate was born to a 36-year-old G1P0 female. History was notable for intravenous drug use, and her urine toxicology screen at delivery of the neonate was positive for fentanyl. Additional testing also revealed a positive hepatitis C infection. The mother admits to the usage of IV fentanyl the day before the presentation to labor and delivery. The pregnancy was also complicated by preterm labor, tobacco use during pregnancy, and delayed presentation to the hospital. Mother reports that she noticed clear vaginal discharge, presumably a rupture of membranes, three days prior to presentation.

Clinical course

Upon arrival at the neonatal intensive care unit, the newborn was extensively examined to determine the extent of genital trauma. Penile skin excoriation, ecchymosis, and necrosis at the tip of the penis appeared to be superficial. Urology determined that conservative management was appropriate, and the neonate was treated with bacitracin ointment to prevent further infection.

The neonate’s overall status remained stable, and recovery was progressing as expected. Recovery was complicated by neonatal abstinence syndrome, requiring the use of scheduled IV morphine, and sepsis with gram-positive bacilli, requiring IV antibiotics.

**Figure 3:**
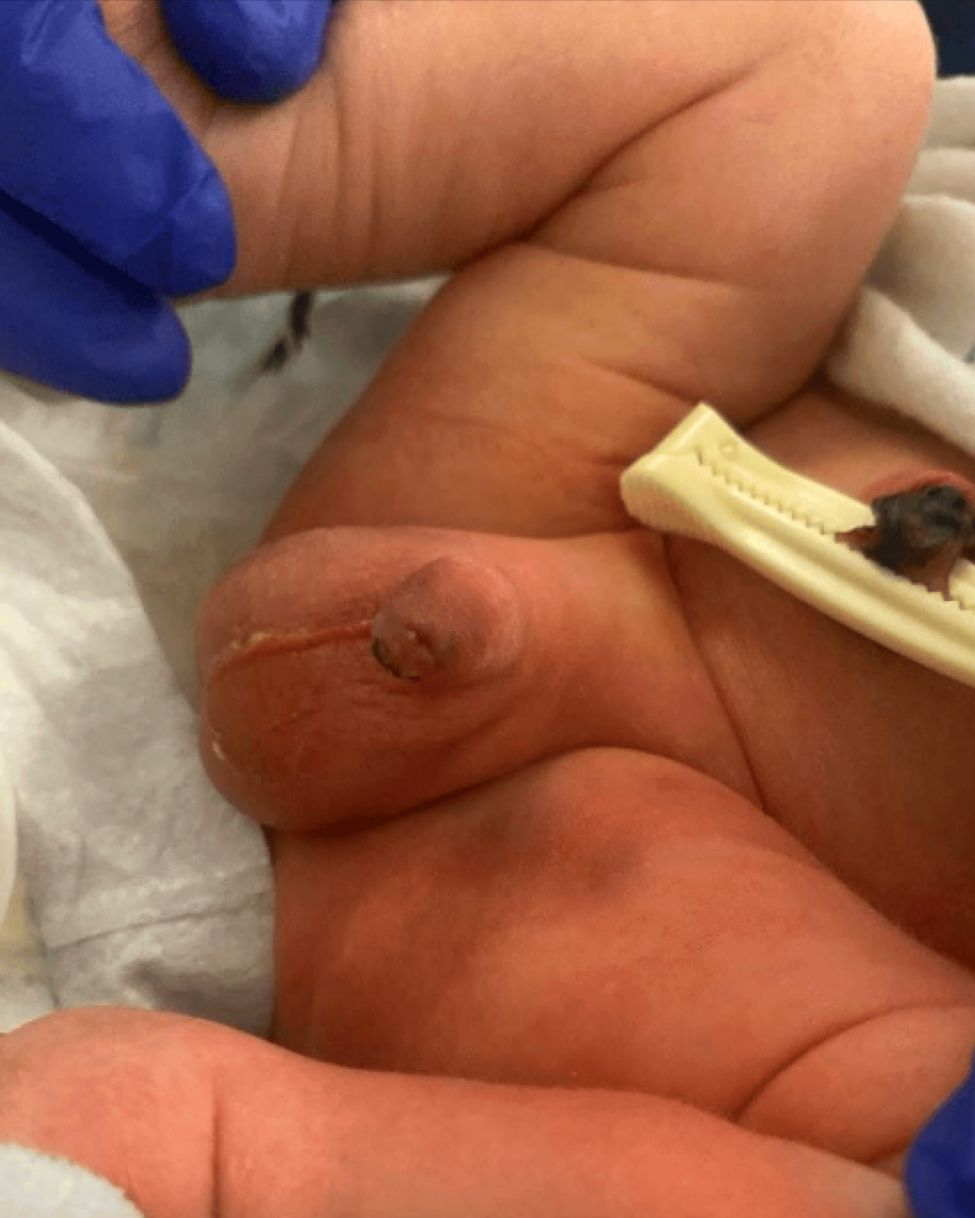
Image of neonate prior to discharge at day 6 of life. Ecchymosis and scrotal sloughing have greatly improved.

Frequent and regular assessment of the injury showed gradual improvement of the penile injury. Feedings were initiated, and the patient demonstrated good suckling reflexes, although there was mild discomfort from the penile injury. Great care was given during diaper changes to minimize additional trauma to the penis due to friction. The penile lesion continued to improve, and on day of life 7, the penile injury appeared to have completely resolved, as seen in Figure 3. No long-term complications from the injury are expected, and the patient was discharged home.

A follow-up visit with an outpatient pediatrician three days later showed a normal, healthy infant. No abnormalities were seen during examination, and the penis appeared to have healed well without complications.

## Discussion

Penile injury in neonates is a rare but recognized complication most often associated with complex deliveries such as breech presentations or operative interventions. As previously documented in a 2004 case report by Finan and Redman, penile injury occurs in approximately 10% of breech presentations [8]. Their case report raised awareness of the potential for trauma to the external genitalia with complicated deliveries.

Injury types may range from superficial skin excoriations and bruising to more severe forms such as penile fracture or urethral trauma. Finan and Redman documented cases of severe ecchymosis and edema of the scrotal tissue, presumably due to mechanical stress during delivery [8]. As in our case, the neonate sustained trauma to the penile prepuce and scrotum as a result of mechanical friction of the genitalia in the birth canal with each contraction, likely secondary to delayed presentation to the labor and delivery unit. Fortunately, the injury was limited to superficial integumentary structures, sparing deeper genitourinary involvement.

Conservative management, including topical antibiotic ointment, was sufficient to prevent infection and promote healing. Finan and Redman managed their patient similarly with scrotal elevation and antibiotic ointment, which also led to resolution [8]. Given the superficial nature of the injury, no long-term sequelae are anticipated. If more extensive injury to the scrotum had occurred, testicular atrophy could occur, as suggested by Finan and Redman. Early recognition, prompt intervention, and close follow-up were instrumental in ensuring optimal recovery and a favorable outcome.

In addition to the promotion of prompt conservative treatment, several maternal factors could have prevented this footling breech presentation and, consequently, adverse pregnancy outcomes. The American College of Obstetricians and Gynecologists recommends universal screening for substance use during prenatal visits [9]. However, due to the patient’s lack of knowledge of being pregnant, she did not receive prenatal care. Risk factors present in the maternal history that could have been optimized, such as smoking and maternal hepatitis C infection, may have reduced the risk of both intrauterine growth restriction and preterm labor, and by extension malpresentation [10-11]. Taken together, unrecognized pregnancy and unaddressed maternal risk factors can culminate in a more difficult delivery with increased risk of obstetric complications.

## Conclusions

Neonatal penile trauma secondary to a frank breech presentation is a rare but possible complication that can occur. The risk is increased with delayed presentation for definitive management with cesarean section, highlighting the importance of early detection and routine prenatal care. The neonate highlighted in the case report fortunately experienced only minor superficial genital injuries that will likely not result in permanent damage with conservative management, as outlined. Close monitoring of injuries is still essential, as testicular atrophy is a potential complication with more severe injuries. As breech deliveries still routinely occur, continued vigilance is needed, as cases such as this one are preventable with routine sonographic evaluation.
